# Shifts in Ecological Dominance between Two Lepidopteran Species in Refuge Areas of Bt Cotton

**DOI:** 10.3390/insects12020157

**Published:** 2021-02-12

**Authors:** José Bruno Malaquias, Danilo Renato Santiago Santana, Paulo Eduardo Degrande, Claudia Pio Ferreira, Elmo Pontes de Melo, Wesley Augusto Conde Godoy, Jéssica Karina da Silva Pachú, Francisco de Sousa Ramalho, Celso Omoto, Alexandre Igor de Azevedo Pereira, Renato Anastacio Guazina

**Affiliations:** 1 Department of Biostatistics, Institute of Biosciences–IBB, São Paulo State University (UNESP), Botucatu 18618-693, Brazil; claudia.pio@unesp.br; 2 Department of Entomology, Federal University of Grande Dourados (UFGD), Dourados 79825-070, Brazil; danilosantana1987@hotmail.com (D.R.S.S.); PauloDegrande@ufgd.edu.br (P.E.D.); renatoaguazina@hotmail.com (R.A.G.); 3 Federal Institute of Mato Grosso do Sul, Ponta Porã 79909-000, Brazil; elmo.melo@ifms.edu.br; 4 Department of Entomology and Acarology, Luiz de Queiroz College of Agriculture (ESALQ), Piracicaba 13418-900, Brazil; wacgodoy@usp.br (W.A.C.G.); jessikapachu@gmail.com (J.K.d.S.P.); celso.omoto@usp.br (C.O.); 5 Biological Control Unit, Embrapa Cotton, Campina Grande 58428-095, Brazil; ramalhohvv@gmail.com; 6 Goiano Federal Institute, Urutaí 75790-000, Brazil; alexandre.pereira@ifgoiano.edu.br

**Keywords:** resistance management, competition, spatial model

## Abstract

**Simple Summary:**

Understanding the reasons that substantiate competitive strategies as a result of selective pressure and their consequences for the dynamics of competitors under specific conditions is one of the key issues in Game Theory. Here, we discuss how the adoption of insecticide control in refuge areas and the occurrence of *Spodoptera frugiperda* and *Helicoverpa armigera* resistance to insecticides could impact the large-scale production of individuals in refuge areas of Bt cotton in the context of competition dynamics. In view of our results, we emphasize the necessity of carefully managing refuge areas of Bt cotton in agroecosystems that have both *S. frugiperda* and *H. armigera*.

**Abstract:**

Competition behavior involving agricultural pest species has long been viewed as a powerful selective force that drives ecological and phenotypic diversity. In this context, a Game Theory-based approach may be useful to describe the decision-making dilemma of a competitor with impacts to guarantee its superiority in terms of ecological dominance or sharing of the food resource with its competitor. In an attempt to elucidate the consequences of competitive dynamics for the ecological dominance of these species in refuge areas of Bt cotton, we conducted a study that was divided into two parts. The first study consisted of an evaluation of interactions involving *Spodoptera frugiperda* (JE Smith, 1797) and *Helicoverpa armigera* (Hübner, 1808) on non-Bt cotton plants in a field trial. In the second study, we explored the data matrix collected in the field to parameterize a model of Cellular Automata (CA) with update rules inspired by Game Theory. Computer simulations were analyzed in hypothetical scenarios involving the application (or not) of insecticides in the refuge areas in combination with the resistance factor of one or both pest species to the insecticides used in the refuge areas. *H. armigera* had superior competitive performance in relation to *S. frugiperda* only at high densities. According to the density-mediated shift in dominance of the species, the resistance of *S. frugiperda* to insecticides is seen as a risk factor for the production of susceptible individuals of *H. armigera* on a large scale in the refuge areas. Additionally, *S. frugiperda* insecticide resistance may potentially impact the resistance evolution of the *H. armigera* population to Bt cotton. Thus, ecological dominance could diverge by the presence of a resistance allele to insecticides with interspecific competition perhaps subordinate to evolutionary processes.

## 1. Introduction

Competition between species has shaped broad-scale patterns of the phenotypic diversity of agricultural pests, and this can be impacted in several ways. One of them is density dependence, which is one of the main factors associated with competition for food resources in a wide variety of insect species, as it directly influences the individual or population fitness of these organisms. In general, density dependence may be regulated by ecological population mechanisms, which lead to shifts in the population structure of species [1]. The nature of these intra- and interspecific interactions can vary according to the evolutionary context and environmental conditions [2]. Experimental and theoretical studies have generally been limited by the lack of methodological approaches to analyze species interactions [3].

The strategic situation in which competitors make different decisions according to the condition of selection pressure may be analyzed with Game Theory [4]. In general, Game Theory formalism is based on competitor behavioral decision making on an individual basis; however, the payoff for each player will depend on the decision made by all competitors who are interacting [5]. The key issue of Game Theory is the understanding of the reasons for decisions given by certain scenarios and their consequences for population fitness under specific conditions [6]. The prisoner’s dilemma is a standard example of Game Theory, which shows why two individuals may not cooperate [4,7]. Game Theory can jointly consider the ecological and evolutionary dynamics of competitors [8]. Despite the frequent use of Game Theory concepts in studies involving cooperative behavior, little attention has been given to the development of models capable of explaining the competition dynamics of pest species in neotropical agroecosystems [9].

*Bacillus thuringiensis* δ-endotoxins (Bt toxins) have been widely used in transgenic cotton due to their toxicity and specificity against important lepidopteran pests from Brazilian cotton agroecosystems, such as *Spodoptera frugiperda* (JE Smith 1797) and *Helicoverpa armigera* (Hübner, 1808) (Lepidoptera: Noctuidae). To delay the evolution of resistance to Bt crops and insecticides, the high dose/refuge strategy has been designed to prevent resistance evolution in insect populations against Bt toxins. This strategy could be effective if the following assumptions are met: resistance alleles to a Bt protein are rare; a Bt protein is consistently produced by a plant at a highly toxic concentration, with resistance alleles that are functionally recessive; and cultivation of refuge areas with non-Bt plants [10]. Refuges of non-Bt crops can be a source of Bt- or insecticide-susceptible individuals that contribute to delaying resistance [11,12]. Ideally, these refuges are planted near Bt crop fields or fields where critical insecticides are used to allow Bt-resistant insects to mate with susceptible insects produced in refuges [13].

A high-dose Bt plant expresses a high concentration of toxin to ensure that at least 95% of the heterozygous individuals carrying one copy of a resistance allele may be controlled [14]. High mortality of the heterozygous of *S. frugiperda* on Bt cotton varieties Wi-deStrike^®^ and TwinLink^®^ has been found in Brazil [15]. However, the intensive planting systems in Brazil have promoted the emergence of a high frequency of individuals resistant to synthetic insecticides and Bt crops. For example, *S. frugiperda* rapidly evolved resistance to the Bt toxin Cry1F produced by Bt cotton and maize [16], Cry1Ab in cotton, maize and soybean [17], and Cry1A.105 in maize [18]. High susceptibility to Cry1Ac of *Helicoverpa armigera* has also been documented in Brazil [10]. However, due to *H. armigera* having low susceptibility to Vip3Aa20, Leite et al. [19] highlight that it is extremely important to monitor the susceptibility of *H. armigera* populations, when crops such as cotton are expressing Vip3Aa20 protein. Although Bt crops can reduce the insecticide sprays to control lepidopteran pests [20,21], on average, in Brazil, it is still necessary to spray insecticides 7 to 9 times in cotton [22]. As a result, *S. frugiperda* and *H. armigera*, in some regions in Brazil, have rapidly evolved resistance to new insecticides, such as diamides [22,23].

*Spodoptera frugiperda* and *H. armigera* are cannibalistic in their larval stage [24]. At the field level, these pests can infest the plant simultaneously during the season and feed on the same cotton structures, such as squares, flower, bolls, and blooms. Thus, they may compete for food resources, exhibiting different behavioral strategies inherent to each species, with intraguild competition involving these two species [25] as a mechanism to guarantee the survival and/or ecological dominance of the one species in the agroecosystem.

The influence of cannibalism and competition between immature *S. frugiperda* and *H. armigera* in situations of density dependence has not been clearly demonstrated. A question of interest to both researchers and cotton farmers in countries with the simultaneous occurrence of *S. frugiperda* and *H. armigera* species would be: What are the competition dynamics of these pests in cotton plants that do not express *Bacillus thuringiensis* (Bt), and which are used as refuge areas for Bt cotton? Based on the assumption that refuge areas are essential for the management of insect resistance to Bt technology, and as they aim at the large-scale production of individuals susceptible to Bt plants [26], a second question would be: How do the competition dynamics of these lepidopterans impact the production of individuals in those refuge areas?

To answer the previous two questions, we have structured this research into two parts. The first part aimed at understanding the intra- and interspecific interaction between *H. armigera* and *S. frugiperda* in non-Bt cotton plants, through biological experiments conducted in the field, whose results were used to parameterize a computer model based on cellular automata. Cellular automata (CA) constitute simple mathematical models, in which space and time are discrete [27]. A regular lattice with a CA in each cell (site or patch) is set following a series of well-defined rules based on a pre-specified neighborhood of the cellular automata interacting in time [28]. Results from the model constituted the second part of our research. The structure of a spatial game model was used in cellular automata described in a formal way and exploring a finite set of competitors. In this case, the larvae are the competitors (players); the interactions involving the competitors are given by the competition strategies in each scenario analyzed and according to the payoff matrix corresponding to the survival rate of the larvae obtained from our intra- and interspecific competition experiments involving *H. armigera* and *S. frugiperda*. Therefore, the competition strategies that are being addressed in our cellular automata are those related to competition among individuals of the same species, and between those of different species.

Taking into account that the evaluation of the consequences of competitive behavior for the population dynamics of *S. frugiperda* and *H. armigera* in refuge areas for Bt cotton technology is of great economic relevance, we focused in our field experiments on the density dependence interactions occurring between competing larvae of *S. frugiperda* and *H. armigera* that could exist within the same non-Bt cotton plant. We also explore, with computer simulations, hypothetical scenarios that were chosen based on possible cases found in Bt cotton agroecosystems, for example, the use of insecticide control with synthetic insecticides in refuge areas [29]. Given the high selection pressure in tropical countries and the impact of the density dependence in resistance evolution, cases of resistance of one or both species to the insecticides used in the refuge areas are expected.

Simulations were carried out in order to explain, from an ecological point of view, the relative dominance of the pests in the analyzed agroecosystems and to contribute to the design of a sustainable management of *S. frugiperda* and *H. armigera* in refuge areas, aiming at the maximum production of Bt-susceptible adults on a large scale, making it possible to delay the resistance evolution of these pests to Bt cotton. Thus, in our simulations, we analyzed the population dynamics occurring with the occupation of the different density state species in the cells (patches) of the cellular automata and, after that, we calculated the relative dominance of each species by each area in five scenarios, as follows: (i) absence of insecticides; (ii) adoption of insecticides without cases of resistance; (iii) adoption of insecticides with population resistance of *S. frugiperda*; (iv) adoption of insecticides with population resistance of *H. armigera*; (v) adoption of insecticides with population resistance of *S. frugiperda* and *H. armigera*.

## 2. Materials and Methods

### 2.1. Intra- and Interspecific Competition in the Field

*Location and Experiment Characterization*: The experiment was conducted in an experimental area located at the Federal University of Grande Dourados (UFGD in Dourados, Mato Grosso do Sul, MS), in the 2017/2018 cotton season, between November 2017 (soil preparation and tillage) and June 2018 (end of the cotton cycle, 154 days after seedlings emergence).

*Insects:* The insects were provided by the Pragas.com company. Both species were reared on artificial diets [30], in a conditioned room under temperature of 25 ± 2 °C, relative humidity of 70 ± 5%, and 12 h of photophase, for more than 5 generations in the absence of selection pressure by insecticides and/or Bt proteins for intra- and interspecific competition studies in the field.

The studies with the isolated species and in combinations were carried out in the field with confinement of the insects in the larval stage, at the R1 reproductive stage of the cotton plants.

Non-Bt cotton plants (FiberMax FM 944GL^®^ BASF) were grown in the field with soil classified, according to Embrapa [31], as Dystrophic Red Latosol. The cotton seeds were sewn by hand in December 2017 with a spacing of 0.90 m in rows and density of eight seeds/m. Fertilizers were applied according to the recommended management for the crop. Weed control was manual and the experimental crop was irrigated when necessary.

The plot consisted of four rows, each row with a length of 4 m. As a useful plot, the central row (2nd or 3rd row) was taken, with 1.0 m at each end border, totaling 7.20 m^2^ of usable area where we selected one single plant (the sample unit). Thus, the larvae of each species were confined in an isolated plant.

The infestations were carried out with *H. armigera* and *S. frugiperda* neonates in a single plant of cotton, with two trials arranged independently, as follows:

Experiment 1—Intraspecific competition with *S. frugiperda* and *H. armigera* species kept isolated, with densities of: 5, 10, 15, and 20 larvae per plant.

Experiment 2—Intra- and interspecific competition involving *S. frugiperda* and *H. armigera* on the same plant, with densities of 5, 10, 15, and 20 larvae of each species per plant.

The neonates were placed with a paintbrush in the cotton squares. Cages were installed on the cotton plants with the following dimensions: 0.70 × 0.70 m wide (at the base) and 1.40 m high, covered with voile Bell-34^®^. Thus, the larvae could not escape and we could avoid natural (external) infestation. We used the Randomized Block Design (RBD) in a 4 × 2 factorial treatment arrangement, with four densities (factor one), two species (factor two), and four blocks = four plants/treatment in total. The evaluations were performed 10 days after the infestation, recording the total number of surviving insects.

*Data analysis*: Two generalized linear models, one model for each experiment, with binomial distribution were fitted to the survival data of *S. frugiperda* and *H. armigera* larvae. The goodness of fit of a statistical model was confirmed using a simulated half-normal plot using the R package hnp [32].

### 2.2. Computer Simulation

#### 2.2.1. Description of the Proposed Model

In the model parameterization, the results of the intra- and interspecific competition experiments involving *H. armigera* and *S. frugiperda* were used. We used two density conditions, “high” and “low”. A density of 5 larvae of each species per cotton plant was considered a “low density” condition, while the “high density” condition was 20 larvae of each insect species per cotton plant. The explored data related to larval survival rate are expressed in the results, and explained in more detail throughout the description of the cellular automata rules.

A two dimension (1000 × 1000 cells) cellular automata model was used in the simulations. After species interaction, each cell can assume one of the three states: “presence of *S. frugiperda*” (species 1), “presence of *H. armigera*” (species 2), or “absence of any of the species”. Parallel update with periodic boundary conditions and the Moore neighborhood with radius one were used.

Each cell in the automata represents a cotton plant. Each time step, *t*, corresponds to a generation of insects. The update rules were:

*(a)Occupied Site*: A cell occupied by species one or two may become empty due to two independent conditions:

*Condition I*. With μ probability (*Natural Mortality* (NM) parameter described in the sensitivity analysis), the occupied site becomes empty, due to the effects of natural mortality factors and/or dispersal (displacement) to other areas.

**Figure 1:**
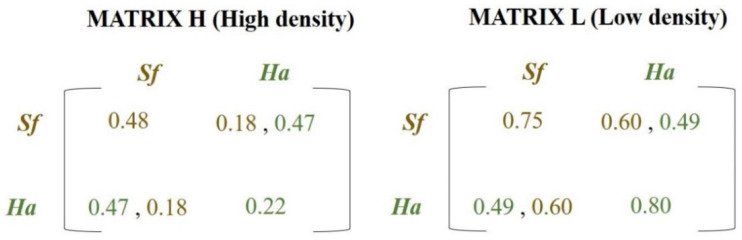
Payoff matrices containing the survival probabilities of *Helicoverpa armigera* (*Ha*) (green values) and *Spodoptera frugiperda* (*Sf*) (brown values). The matrices represent the payoffs given the inter + intraspecific interactions (principal diagonal) or only interspecific interactions (secondary diagonal) for states with high population densities (**MATRIX H** (**High density**)) or low population densities (**MATRIX L** (**Low density**)). These results were obtained in the biological experiments and they are expressed in Figure 2A–C (Section 3). (For a color version of this figure, the reader is referred to the web version of this article.).

*Condition II*. If the situation described in item 1 does not occur, this site may be empty with probability *θ*, due to intra- and/or interspecific competition which was calculated as follows: First, the number of occupied sites in the Moore neighborhood (eight closest neighbors) was calculated. If this number is greater than or equal to *T* (threshold previously defined), which represents a density state, then **Matrix H** (High Density Matrix) was selected (Figure 1); otherwise, data from **Matrix L** (Low Density Matrix) (Figure 1) were used. Subsequently, the encounter probability between two competing species was calculated, which also depended on the number of sites occupied by each species in the Moore neighborhood. If the species were on the same plant during the larval stage, the probability for the survival of that phase was given by the intra- and interspecific interactions (secondary diagonals of **Matrices H** or **L**) (Figure 1). Otherwise, the survival probability was given only by intraspecific interactions (principal diagonals of **Matrices H** or **L**) (Figure 1). The parameter, *θ*, previously defined, was one minus the survival probability.

*Condition III*. If the conditions *I* and *II* were not met, then the site was still occupied. An example summarizing the rules mentioned above is provided below:

If the individuals’ frequency in the local neighborhood is greater than 0.50 (arbitrary density state—**MATRIX H**, Figure 1) and the probability of encounter on the same site between these species is not met, the probabilities of larval survival of *S. frugiperda* and *H. armigera* are 0.48 and 0.22 (principal diagonal of **MATRIX H**—Figure 1), respectively. In the case of encounter between these two species, the larval survival probability of *S. frugiperda* is 0.18, while that of *H. armigera* is 0.47 (secondary diagonal of **MATRIX H**—Figure 1).

*(b) Empty Site*: An empty site may become occupied with probability *W*, due to the insect oviposition in the Moore neighborhood. The probability *W* was given by the relative fitness (*f_1_* and *f_2_*).

We estimated the relative fitness based on the reproductive success of each insect species on reaching the adult stage. To calculate reproductive success, the number of insects of each species in a Moore neighborhood was multiplied by their respective reproductive capacity (*R_o_*).

The reproductive capacity considered in the model was based on the number of eggs and neonate hatch rate, according to biological data on *H. armigera* and *S. frugiperda* collected in cotton by Gomes [33] and Barros et al. [34], respectively.

The probability of species one (*S. frugiperda*) ovipositing is given by *f_1_*. If species one does not oviposit, then the probability of oviposition of species two (*H. armigera*), given by *f_2_*, is tested. If none of the species oviposit, then the site remains empty.

*Description of the Scenarios Used in the Simulations:* The relative dominance as a function of the time of each species by area and which involves the proportion of individuals of each species in relation to the total was calculated in five scenarios, as follows:

- Non-adoption of insecticides.
- Adoption of insecticides without cases of resistance.
- Adoption of insecticides with population resistance of *S. frugiperda* to the used insecticide.
- Adoption of insecticides with population resistance of *H. armigera* to the used insecticide.
- Adoption of insecticides with population resistance of *S. frugiperda* and *H. armigera* to the used insecticide.

The insecticide was sprayed only when one of the species reached the arbitrary control level (parameter CL—see Table 1) of 20%. We checked in each time step if the number of cells had reached the proportion of 0.20 for at least one of the species in relation to the total number of cells.

**Figure 2:**
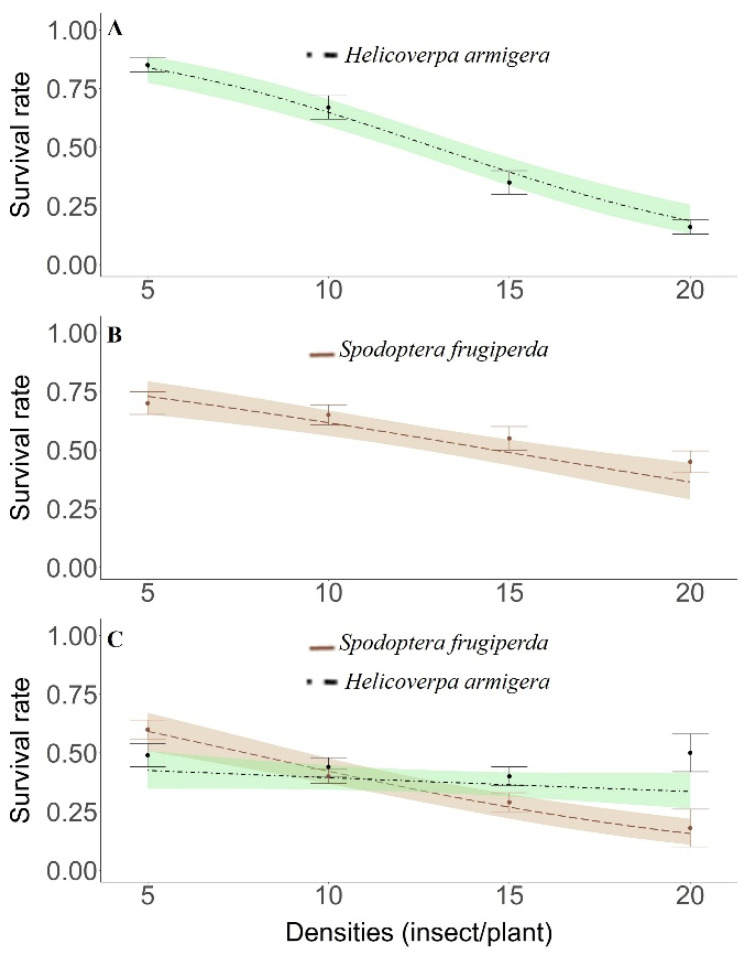
In-field survival rate (mean ± SE) of *Spodoptera frugiperda* and *Helicoverpa armigera* larvae isolated (intraspecific competition only) and combined (intra- and interspecific competition). Dashed curve corresponds to the observed data, while the colored areas are the confidence regions (95% CR) predicted by the generalized linear model with binomial distribution. (For a color version of this figure, the reader is referred to the web version of this article.). (**A**) Only *H. armigera*; (**B**) Only *S. frugiperda*; (**C**) *S. Frugiperda* + *H. armigera*.

The Cellular Automata (CA) model was parametrized with the data provided using a logistic regression model (Figure 2) with Bayesian inference adjusted to the experimental data collected in the field experiments. The rules of the CA model followed prisoner’s dilemma (Game Theory). The initial number (absolute frequency) of individuals was 500 individuals of each species or a relative dominance of 0.50.

The mortality probability from the use of insecticide of 0.99 was considered for larvae surviving the effects of natural mortality or displacement factors (dispersal to other agroecosystems). The use of insecticide did not alter any other rules of the automata. For the scenarios of resistance occurrence, we assumed that the population was in Hardy–Weinberg equilibrium, and that the initial frequency of insecticide-resistant individuals adopted in the simulations was 0.0625.

A description is presented in Table 1 of all the parameters, as well as their adopted values in the simulations and the range explored in the sensitivity analysis.

#### 2.2.2. Parameter Sensitivity Analysis

We measured the impacts of fluctuations in parameters of our model on the outputs. The sensitivity analysis is based on a unique Principal Component Analysis (PCA). The sensitivity indices were estimated based on the weighted participation of each parameter in the main components, thus calculating the generalized sensitivity index, according to the method described by Lamboni et al. [36] in the R package multisensi [37]. The parameter values for sensitivity analysis were obtained through uniform distribution (Table 1).

The ranges of uniform distribution for each parameter were given by a rate of change of 25% of the original value for each parameter. A detailed description of the parameter values and the range used in the sensitivity analysis is shown in Table 1.

## 3. Results

### 3.1. Intra- and Interspecific Competition in the Field

A linear decline in the survival rate was found for both species, *H. armigera* and *S. frugiperda*, kept in non-Bt cotton plants either in isolation (intraspecific competition) or combined (interspecific competition) in the evaluated density range (Figure 2a–c). Independently of *S. frugiperda* larvae density, kept in isolation, there was competition between the larvae of this species, with the survival rate ranging near to 0.75 and 0.50 in the densities of 5 and 20 larvae per plant, respectively (Figure 2b). *H. armigera* had maximum and minimum survival rates of 0.80 and 0.22 at densities of 5 and 20 larvae per plant, respectively, in the same isolated confinement bioassay (Figure 2a).

In the intra- and interspecific interaction assay involving *S. frugiperda* versus *H. armigera* larvae, the species with the highest survival in the lowest and intermediate densities was *S. frugiperda*. On the other hand, at a density of 20 larvae of each species, the greater predominance was of *H. armigera* larvae (survival rate > 0.45%) in relation to *S. frugiperda* (survival rate < 0.20) (Figure 2c).

### 3.2. Computer Simulation

**Figure 3:**
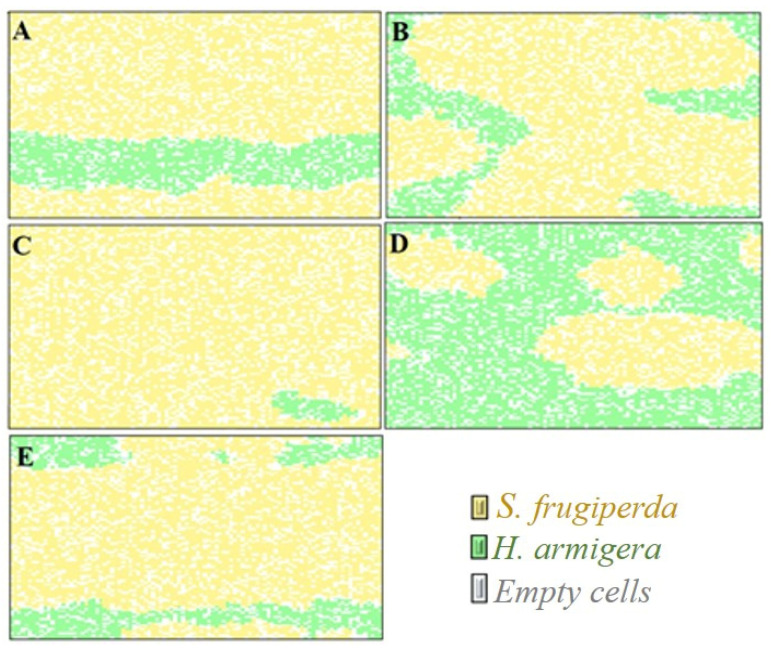
Visual representation of the occupation of patches by *S. frugiperda* and *H. armigera* after 80 time steps in the following conditions: (**A**): absence of insecticide control. (**B**): insecticide control and absence of cases of insecticide resistance. (**C**): insecticide control and resistance of *S. frugiperda* to the insecticide. (**D**): insecticide control and resistance of *H. armigera* to the insecticide. (**E**): insecticide control and resistance of *S. frugiperda* and *H. armigera* to the insecticide. (For a color version of this figure, the reader is referred to the web version of this article.)

The simulations revealed that, over the course of 80 generations (Figure 3a–d), *S. frugiperda* was the dominant species in all scenarios with exception only in the scenario of adopting insecticide control and resistance of only *H. armigera* to the insecticide, where there was dominance of *H. armigera* (Figure 3d). The greatest negative impacts on the population dynamics of *H. armigera* were with the use of insecticide control and with the occurrence of *S. frugiperda* resistance to the insecticide, providing the formation of small islands of its competitor (Figure 3c). Similarly, despite the resistance of both species to the insecticide used in the refuge areas (Figure 3e), the patch competition between larvae ne-gatively impacted the *H. armigera* population.

**Figure 4:**
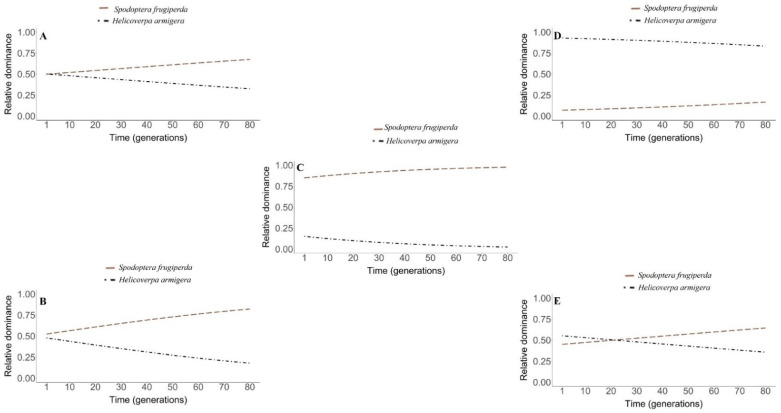
Dynamics of temporal evolution of the ecological dominance of *S. frugiperda* (light salmon long dashed line) and *H. armigera* (black dotted line) in the following conditions: (**A**): absence of insecticide control. (**B**): insecticide control and absence of cases of insecticide resistance. (**C**): insecticide control and resistance of *S. frugiperda* to the insecticide. (**D**): insecticide control and resistance of *H. armigera* to the insecticide. (**E**): insecticide control and resistance of *S. frugiperda* and *H. armigera* to the insecticide. (For a color version of this figure, the reader is referred to the web version of this article.)

In the analysis on the time evolution of the patch occupation by the competitors, the lowest population impacts were observed for *H. armigera* when there was no adoption of insecticide control in the refuge areas, among the scenarios with predominance of *S. frugiperda* (Figure 4a).

In the absence of insecticide control, the curves of competing species have the same intercept; in other words, the same origin of ecological dominance evolution (Figure 4a).

In the scenario of insecticide control with no cases of resistance for both species, the results from our modelling revealed that despite the small difference, the curves of competing species do not have a common intercept (Figure 4b). In addition, the ecological dominance was increasing and significantly higher in *S. frugiperda* in relation to *H. armigera*, since there was no overlapping of the curves at any time (Figure 4b).

In the adoption of insecticide control and the occurrence of resistance for *S. frugiperda*, the simulations revealed a high dominance of this species in relation to *H. armigera* over the generations. Although there was no risk of extinction of *H. armigera*, its relative do-minance was estimated to be less than 10% after 70 steps of time (Figure 4c).

The incorporation of the resistance of *H. armigera* and the absence of this genetic trait in *S. frugiperda* resulted in the ecological dominance of the first species. Nevertheless, the predicted curves of ecological dominance of competing species over time were contrasting; therefore, in *S. frugiperda*, the response was increasing, while in *H. armigera*, it was decreasing (Figure 4d).

In the scenario with the use of insecticide control and resistance of both species in the refuge areas, we obtained a greater dominance of *H. armigera* in relation to *S. frugiperda* in the interval that precedes 18 initial time steps (generations). After this, there was an overlap of the curves, followed by a shift in the ecological dominance; therefore, after this period, the dominant species was *S. frugiperda* (Figure 4e).

### 3.3. Sensitivity Analysis

Based on the generalized sensitivity index (*GSI*), we verified the following decreasing order of sensitivity variation on the population dynamics for both insect species to model parameters:

*IF* > *R_o_Sf* > *AE* > *NM* > *β*_11_ > *α*_22_ > *β*_12_ > *β*_22_ > *R_o_Ha* > *α*_12_ > *CL* > *β*_21_ > *α*_21_ > *α*_11_

**Figure 5:**
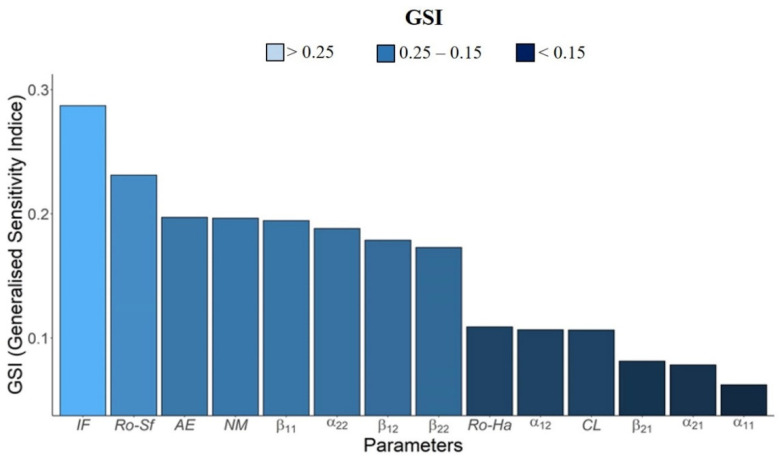
Generalized sensitivity index based on principal component analysis, for the following parameters: AE: Adult emergence. IF: Initial frequency. R0-Sf: Net reproduction rate of *S. frugiperda*. R0-Ha: Net reproductive rate of *H. armigera*. NM: Natural mortality. CL: Control level. α11: Survival of *S. frugiperda* in intraspecific competition at low densities. α12: Survival of *S. frugiperda* in intra- and interspecific competition at low densities. α21: Survival of *H. armigera* in intra- and interspecific competition at low densities. α22: Survival of *H. armigera* in intraspecific competition at low densities. β11: Survival of *S. frugiperda* in intraspecific competition at high densities. β12: Survival of *S. frugiperda* in intra- and interspecific competition at high densities. β21: Survival of *H. armigera* in intra- and interspecific competition at high densities. β22: Survival of *H. armigera* in intraspecific competition at high densities. (For a color version of this figure, the reader is referred to the web version of this article.).

The parameter most sensitive to variations in the population dynamics of the species *S. frugiperda* and *H. armigera* is the initial frequency of individuals, followed by the net reproduction of *S. frugiperda*, adult emergence, and natural mortality. On the other hand, the parameter with the lowest *GSI* is the survival rate of *S. frugiperda* in conditions of intraspecific competition at low densities (Figure 5).

## 4. Discussion

The interspecific competitive performance of *S. frugiperda* is higher in relation to *H. armigera*, except under conditions of high larval densities. Therefore, this result reveals that density can mediate shifts in ecological dominance between these two species, and that *H. armigera* is more responsive at high densities in the interspecific interaction with *S. frugiperda*. We try to explain the greater survival of *H. armigera* in relation to *S. frugiperda* in biological trials occurring at the highest density tested, providing four possible reasons.

The first reason may be supported by the intraspecific competition effect. This fact was verified independently of the density in isolated studies either with *S. frugiperda* or *H. armigera* larvae. In interspecific competition, the cannibalism could be more intense for *S. frugiperda* specimens in high densities in relation to *H. armigera*, which may have favored a greater survival rate of *H. armigera* when compared to that species. There is evidence that third instar *S. frugiperda* larvae increase intraspecific competition when food is scarce [38]. However, in other cases, the interaction of the diet with the opportunity to cannibalize is not important to *S. frugiperda*, indicating that the reduction in survival associated with the presence of members of the same species was uniform without relation to food availability [39]. We infer that the second reason may be related to the adverse effect of competition between species, or even that the attack and attempt to ingest the competitor may cause injury to the larvae, consequently affecting the development of the pest or possibly leading to death. In addition, food scarcity in this case influences the consumption of a greater number of dead bodies of larvae in plants, which may favor the dissemination of microorganisms, such as fungi, viruses, and bacteria that are important biological control agents for the regulation of these pest populations.

The intra- and interspecific competition of larvae depends on the instar and, therefore, the stage of larval development [24,39,40,41]. Thus, we propose that the third reason could be due to the faster development of *H. armigera* larvae, promoting larger larvae in a short time and being more competitive in relation to *S. frugiperda* in the conditions of higher densities, because the consumption of its competitor can promote the intake of readily digestible and metabolizable food resources. The last reason could be linked to the intensification of the more aggressive behavior of *H. armigera* when the larvae are subjected to the highest densities and combined with *S. frugiperda*, because the proximity between them is considered one of the most important components to increase insect aggressiveness [42].

Computer simulations allowed us to infer that when the areas do not receive insecticide control to regulate the larval population, *S. frugiperda* is the dominant species; nevertheless, this scenario of not adopting insecticide control appears to be the most promising for the maintenance of the two lepidopteran species, and the closest to a sustainable program for resistance to Bt cotton. This above assumption is based on the fact that a smart program for managing insect resistance to plants that express Bt, with structured refuges, must be designed in order to enable the production of a large number of susceptible adult insects with minimal economic costs and logistical impact for farmers [42].

The simulations demonstrated that when insecticide control is used for population regulation of these pests, the population dynamics of *H. armigera* is negatively affected, and its impact is even more intensified with the occurrence of *S. frugiperda* resistance to the insecticide used in refuge areas. For a refuge to be sufficiently effective to delay the evolution of insect resistance to Bt toxins, it is important to maximize the availability of susceptible adult insects [43], following the rule of production of at least 500 susceptible adults to a resistant adult [44]. Faced with the necessity for large-scale production of susceptible adults, our simulations highlight the importance of a sustainable management of populations of both *S. frugiperda* and *H. armigera* in refuge areas; in other words, with minimal use, and only when necessary, of insecticide.

The use of insecticides should be associated with proactive resistance management to insecticides used in refuge areas, especially for *S. frugiperda*. Our simulation results suggest that insecticide-free refuge areas favor the population expansion of *H. armigera*, and this is probably due to the influence of the density dependence, allowing *H. armigera* to be rewarded by a higher payoff due to the fact that this species has greater interspecific competitive capacity in relation to *S. frugiperda* under conditions of high density, and in addition, increasing the probability of exploiting patches not occupied by its competitor. The technologies of insecticide spray used currently rarely promote total mortality of the target insects [45], a factor incorporated in our computational model. Even when *H. armigera* larvae survive the insecticide spray, it was possible to infer that there is a low competitiveness of *H. armigera* by patches in the simulated refuge areas. This possibly adaptive density-dependent behavior strategy may be likely mediated by the reduction in cooperative interactions when there are few individuals [46].

In contrast to the lower population performance of *H. armigera* when the insecticide resistance of its competitor occurs, the greatest competitiveness of *H. armigera* at higher densities also allows us to reflect on the higher probability of population expansion of *H. armigera* in an agro-ecosystem with the occurrence of resistance of this species to insecticide and absence of resistance from its competitor, although with less population impacts for *S. frugiperda*. Such evidence motivates further studies involving the competition of these insects in a phenotypic context; that is, research involving the genotypes of *S. frugiperda* and *H. armigera* that are resistant, heterozygous, and susceptible to insecticides and/or Bt plants subjected to various conditions of selection pressure. Knowledge about the competitive potential of these insects in this phenotypic context will allow more accurate inferences to be made about the consequences of the competitive dynamics both for the evolution of the insects’ resistance to the insecticides used in refuge areas and for Bt plants. The natural predation could also provide efficient resistance management with high refuge size because of strong density-dependent mortality [47]. Therefore, to meet the concept of sustainable management in refuge areas, experimental and modelling studies considering other control tactics available to manage lepidopteran species and other pest species, such as applied biological control [48], may be more pertinent.

In conclusion, the results from our biological experiments show a density depen-dence for the survival of *H. armigera* and *S. frugiperda*, when they are kept on cotton plants in isolation or in combination. *S. frugiperda* has a higher competitive performance compared to *H. armigera*, except in the condition of the highest density. Computer simulations allowed us to infer that the resistance of one of the species to the insecticides used in the areas of the refuge of Bt cotton negatively impacts the potential for population expansion of insects, and consequently, the abundance of these individuals, although *S. frugiperda* proves to be the species least sensitive to the insecticide resistance factor of its interspecific competitor. Therefore, according to the impact mentioned in the population structure of competing individuals and the probable reduction in the production of susceptible individuals, it is suggested that programs for managing the resistance of the studied species to Bt cotton may also adopt integrated and sustainable practices to avoid the resistance of species to the insecticides used in structured refuges. Thus, the adoption of such practices may, in addition, delay the evolution of insect resistance to Bt cotton with maximum production of Bt-susceptible individuals aimed in refuge areas of Bt cotton.

## Figures and Tables

**Table 1:** Description values and parameters used in the simulations (S) of the model and in the sensitivity analysis (SA).

Parameter	Description	Value-S ^1^ (Value-AS) ^2^	Reference
*AE*	Adult emergency	0.86 (0.65–1.00)	
Ʊ	Encounter rate between species 1 and 2	*	-
*T*	Threshold for defining the state (high or low) of density	0.50 (***)	**
*CL*	Threshold for decision making for insecticide control	0.2000 (0.15–0.25)	-
*R_o_Ha*	Reproductive capacity (neonates/female) of *Helicoverpa armigera*	725.06 (543.80–965.33)	Gomes et al. [33]
*R_o_Sf*	Reproductive capacity (neonates/female) of *Spodoptera frugiperda*	400.00 (300.00–500.00)	Barros et al. [34]
*W_Ha_*	Relative fitness of *H. armigera*	*	-
*NM*	Probability associated with mortality factors ^#^	0.25 (0.19–0.31)	Varella et al. [35]
*W_Sf_*	Relative fitness of *S. frugiperda*	*	-
*IF*	Initial number (absolute frequency) of *H. armigera* and *S. frugiperda*	500 (375–625)	
*α_11_*	Survival of *S. frugiperda* in intraspecific competition at low densities	0.63 (0.47–0.79)	****
*α_12_*	Survival of *S. frugiperda* in intra- and interspecific competition at low densities	0.58 (0.44–0.73)	****
*α_21_*	Survival of *H. armigera* in intraspecific competition at low densities	0.46 (0.35–0.58)	****
*α_22_*	Survival of *H. armigera* in intra- and interspecific competition at low densities	0.90 (0.68–1.00)	****
*β_11_*	Survival of *S. frugiperda* in intraspecific competition at high densities	0.31 (0.23–0.39)	****
*β_12_*	Survival of *S. frugiperda* in intra- and interspecific competition at high densities	0.11 (0.08–0.14)	****
*β_21_*	Survival of *H. armigera* in intraspecific competition at high densities	0.38 (0.29–0.48)	****
*β_22_*	Survival of *H. armigera* in intra- and interspecific competition at high densities	0.16 (0.12–0.18)	****

^#^ Mortality factors by natural enemies and/or in case of dispersal. ^1^ Value of the parameter used in the simulations. ^2^ Values of the parameters. * Random, according to the density of individuals of species one and two in the Moore neighborhood of radius 1 (eight closest neighbors). ** Arbitrary value. *** Parameter not used in the sensitivity analysis. **** Data collected in biological experiments.

## Data Availability

The data presented in this study are available in this article.
